# Supplementary material for: The interactome of intact mitochondria by cross-linking mass spectrometry provides evidence for coexisting respiratory supercomplexes
Source: Mol Cell Proteomics. 2017 Dec 8;17(2):216–32. doi: 10.1074/mcp.RA117.000470 (PMC5795388; doi:10.1074/mcp.RA117.000470)
Supplement: Supplemental Data [file supp_RA117.000470_133922_0_supp_23978_fzffwf.zip › spectra_annotation/mito_DR_spectra_annotation/430-1-10-1-13-1.pdf]

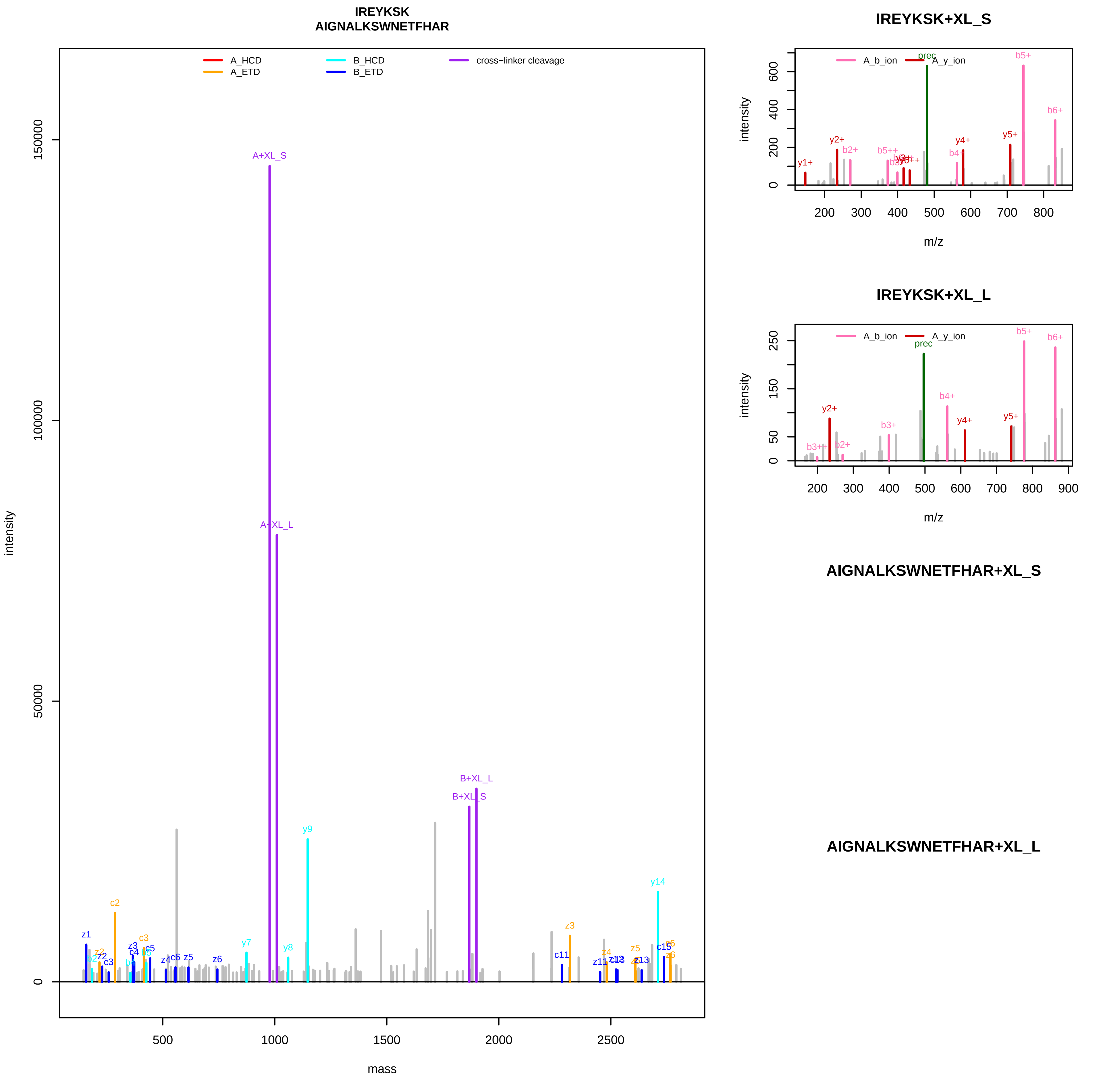
IREYKSK
AIGNALKSWNETFHAR
A_HCD
A_ETD
B_HCD
B_ETD
cross−linker cleavage
A+XL_S
A+XL_L
B+XL_L
B+XL_S
y9
y14
y7
y8
c2
c3
z3
c4
c5
z1
z2
c6
z5
z6
z4
c11
z11
c13
z13
c15
z5
z6
intensity
mass
0
50000
100000
150000
500
1000
1500
2000
2500
IREYKSK+XL_S
A_b_ion
A_y_ion
prec
b5+
b6+
y1+
y2+
b2+
b5++
y4+
y5+
b4+
intensity
m/z
0
200
400
600
200
300
400
500
600
700
800
IREYKSK+XL_L
A_b_ion
A_y_ion
prec
b5+
b6+
b4+
y2+
b3+
y4+
y5+
b3++
b2+
intensity
m/z
0
50
150
250
200
300
400
500
600
700
800
900
AIGNALKSWNETFHAR+XL_S
AIGNALKSWNETFHAR+XL_L
